# Supplementary material for: The genome sequence of the 24-spot ladybird, Subcoccinella vigintiquattuorpunctata (Linnaeus, 1758) (Coleoptera: Coccinellidae)
Source: Wellcome Open Res. 2025 Dec 23;10:698. [Version 1] doi: 10.12688/wellcomeopenres.25409.1 (PMC12895099; doi:10.12688/wellcomeopenres.25409.1)
Supplement: Peer review history

# Reviewer report: Reviewer response for version 1

**DOI:** 10.21956/wellcomeopenres.27995.r146128

**Published:** 2026-02-18

**Authors:** Mario Stanke

**Affiliations:** 1 University of Greifswald, Greifswald, Germany

The paper introduces the genome assembly of the 24-spot ladybird (ladybug). The generated sequencing data includes PacBio HiFi reads, Hi-C data for scaffolding, and whole-organism RNA-Seq from Illumina. The paper largely follows a standardized template for Data Notes on genome assemblies. The results are useful, and plausible to me.

 The presented assembly comfortably satisfies the current thresholds formulated by the EBP standards committee: the N50 exceeds 1 million bp, most of the sequence is assigned to chromosomes, and the estimated per-base error rate is below 1/10000.

 Although the genome was not annotated, the RNA-Seq is a good resource to either create or assess a genome annotation in the future. Regarding the Hi-C contact map in Figure 3, no specific off-diagonal points are visible, only a barely visible background hue. It would be helpful to add a sentence explicitly describing the nature of this off-diagonal signal.

Are sufficient details of methods and materials provided to allow replication by others?

Yes

Is the rationale for creating the dataset(s) clearly described?

Yes

Are the datasets clearly presented in a useable and accessible format?

Yes

Are the protocols appropriate and is the work technically sound?

Yes

Reviewer Expertise:

bioinformatics method development

I confirm that I have read this submission and believe that I have an appropriate level of expertise to confirm that it is of an acceptable scientific standard.

# Reviewer report: Reviewer response for version 1

**DOI:** 10.21956/wellcomeopenres.27995.r146131

**Published:** 2026-02-11

**Authors:** Andres Mariano Alonso

**Affiliations:** 1 Universidad Nacional de San Martín (CONICET-UNSAM), Chascomús, Argentina

Crowley et al. describe the genome assembly of the 24-spot ladybird ( *S. vigintiquattuorpunctata*). The metrics for the presented assembly are adequate, although the authors previously presented an assembly for a similar organism with better performance (10.12688/wellcomeopenres.17346.1). Could these differences be related to genome size or complexity? In this sense, the standard pipeline implemented here could be improved and adapted for the analyzed organism. I suggest the authors add a sentence discussing this in the main text.

 The major issue I observed is related to the contact map (Figure 3). The authors describe features in the main text, but these are not clearly observable in the figure, particularly the TADs. The authors need to revise the presented information and provide a better figure so that the claimed scaffolding is visible to the readers.

Are sufficient details of methods and materials provided to allow replication by others?

Yes

Is the rationale for creating the dataset(s) clearly described?

Yes

Are the datasets clearly presented in a useable and accessible format?

Yes

Are the protocols appropriate and is the work technically sound?

Yes

Reviewer Expertise:

Computational Biology, Bioinformatics

I confirm that I have read this submission and believe that I have an appropriate level of expertise to confirm that it is of an acceptable scientific standard.

# Reviewer report: Reviewer response for version 1

**DOI:** 10.21956/wellcomeopenres.27995.r143212

**Published:** 2026-01-21

**Authors:** Panagiotis Ioannidis

**Affiliations:** 1 Foundation for Research & Technology - Hellas, Crete, Greece

This paper describes the sequencing and assembly of the genome of the beetle Subcoccinella vigitiquattuorpunctata.

 The genome sequencing methodology is the standard one used in all the DToL projects (HiFi + Hi-C + RNAseq). The Hifiasm genome assembler is the tool producing the best genome assemblies from Hifi data. QC is done also using the state of the art (BUSCO + Merqury + Blobplots). The resulting genone assembly is one with a good BUSCO score, QV score and k-mer completeness.

 However, the total number of scaffolds is relatively high (n = 256), and even the vast majority of the genome sequence is assigned to one of the 15 chromosomal level scaffolds, I've seen much better assemblies. This is very obvious in the Blob plot where there are two "clouds" of small scaffolds which on average have higher coverage than the chromosomal scaffolds and they also have a different GC content (both higher and lower than the 32-34% for the chromosomal scaffolds). The authors must comment on the origin of these extra scaffolds, and why are there so many of them?

 Also, the Hi-C contact map (figure 3) only contains the diagonal. How is this possible? Why are there no TADs? I'm suspecting some kind of technical error in the processing of the Hi-C data. Most probably this figure needs to be re-plotted and replaced.

Are sufficient details of methods and materials provided to allow replication by others?

Yes

Is the rationale for creating the dataset(s) clearly described?

Yes

Are the datasets clearly presented in a useable and accessible format?

Yes

Are the protocols appropriate and is the work technically sound?

Yes

Reviewer Expertise:

insect genomics, bioinformatics

I confirm that I have read this submission and believe that I have an appropriate level of expertise to confirm that it is of an acceptable scientific standard.
